# Urethral hemangioma: How to investigate as a cause of hematuria after male sexual activities

**DOI:** 10.1002/iju5.12646

**Published:** 2023-09-24

**Authors:** Kimiyasu Ishikawa, Haruaki Sasaki, Hideaki Shimoyama, Motoki Yamagishi, Hiroo Sugishita, Yu Hashimoto, Yuki Ichimura, Ayana Doguchi, Kazuhiko Oshinomi, Jun Morita

**Affiliations:** ^1^ Department of Urology Yokohama Shin‐midori General Hospital Yokohama Japan; ^2^ Department of Urology Showa University Fujigaoka Hospital Yokohama Japan; ^3^ Department of Urology Showa University Hospital Tokyo Japan

## Abstract

**Introduction:**

Urethral hemangioma is an extremely rare occurrence and is not typically considered a common cause of hematuria. Since 2000, only 22 male cases have been reported.

**Case presentation:**

A 45‐year‐old man presented with recurrent painless gross hematuria and the passage of blood clots after ejaculation. The patient underwent a transurethral resection of a 6‐mm hemangioma. This isolated sessile lesion was situated between the distal end of the verumontanum and the external sphincter, following an induced erection. The patient remained asymptomatic during the 1‐month follow‐up visit.

**Conclusion:**

This study included the assessment of patient symptoms, diagnoses, and treatments and the literature review of 22 patients. We propose that relaxation of the external urethral sphincter muscle under general anesthesia and artificially inducing an erection can aid in the identification of urethral hemangiomas near the verumontanum during cystourethroscopy.

**Keynote message**

Intermittent hematuria is the most common symptom of urethral hemangioma, usually devoid of pain, but can occasionally increase in intensity. Hematospermia may also manifest as a symptom on rare occasions. During cystourethroscopy, artificially inducing an erection through the relaxation of the external urethral sphincter muscle under general anesthesia is useful for identifying hemangiomas near the verumontanum.

## Introduction

Urethral hemangioma is an extremely rare benign vascular tumor and is typically not the cause of hematuria. The most common symptoms associated with urethral hemangioma include indolence in the majority of cases, while intermittent hematuria, albeit infrequent, can be significant in quantity. Regarding the treatment of urethral hemangioma, approaches such as thermo‐ or laser coagulations have been proposed for smaller lesions, while surgical interventions are recommended for larger lesions.

In this study, we demonstrate our experience with an endoscopic resection surgery for urethral hemangioma, particularly in cases involving urinary retention due to post‐penile erectile hematuria.

## Case presentation

A 45‐year‐old man with an unremarkable medical history and no prior surgeries has been consistently experiencing painless gross hematuria and discharge of blood clots following ejaculation since June 2020. He did not complain of hematospermia. Three months following the symptom onset, he received a medical examination at a urology clinic due to the presence of gross hematuria and urinary retention. Magnetic resonance imaging and cystourethroscopy could not confirm the cause of hematuria. Subsequently, for approximately 9 months, he remained unaware of hematuria cases.

**Fig. 1:**
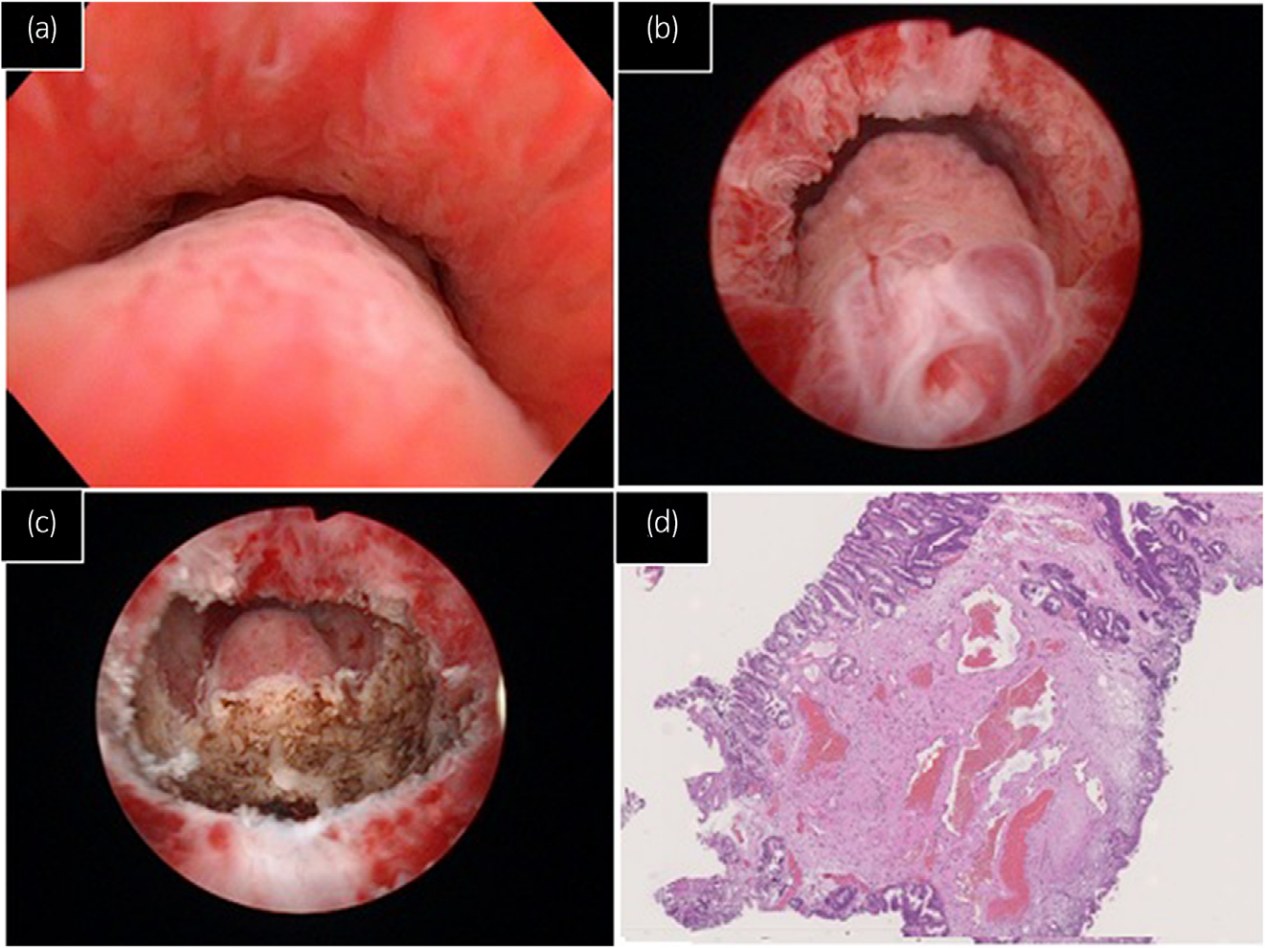
(a) shows the presence of urethral hemangioma, following an induced erection. (b) shows the presence of urethral hemangioma, following an induced erection under general anesthesia. (c) shows the presence of urethral hemangioma after transurethral resection. (d) Histological examination revealed cavernous hemangioma with collection of irregularly dilated vessels in the stromal layer below the epithelium (HE × 400).

In June 2021, the patient was referred to our clinic due to a recurrence of gross hematuria and urinary retention after ejaculation. His physical vital signs were within normal ranges, and both his abdominal and external genital areas were normal. The diameter of the urethral opening was also within the normal range. Laboratory analyses of midstream urine, as well as biochemical, hematological, and coagulation parameters, yielded normal results. Abdominal and pelvic ultrasound and computed tomography scans showed no abnormalities. We performed a procedure wherein we artificially induced an erection by injecting alprostadil alfadex into the corpus cavernosum after securing the base of the penis. Subsequently, cystourethroscopy was performed. The examination revealed the presence of a 6‐mm isolated sessile lesion with varicose veins between the distal end of the verumontanum and the 6 o'clock position of the external sphincter (Fig. 1a). No lesions were detected within the bladder. As the patient's symptoms had resolved at this point, no treatment was administered.

In January 2023, the patient was re‐examined due to the recurrence of gross hematuria after erections. Cystourethroscopy conducted after inducing an artificial erection yielded results consistent with the previous observation. However, a transrectal power Doppler sonography revealed a strong blood flow near the apex of the prostate gland. In March 2023, we excised the lesion while the patient was under general anesthesia. Artificially inducing an erection enabled us to clearly identify an enlarged vascular lesion with varicose veins between the verumontanum and external sphincter (Fig. 1b). Transurethral resection was performed, extending the excision close to the verumontanum (Fig. 1c). Following the surgery, a Foley catheter was retained for 4 days. Histopathological examination revealed that structures comprised an anastomosis network of vascular spaces of varying sizes, lined with endothelial cells and filled with blood. This observation aligned with the characteristics of urethral hemangioma (Fig. 1d). No signs of malignancy were detected. At the follow‐up visit after 1 month, the patient was asymptomatic (no hematuria after ejaculation), and cystoscopy did not detect any new lesions.

## Discussion

Urethral hemangioma is an extremely rare benign vascular tumor and is not a common cause of hematuria. Since 2000, only 23 male cases, inclusive of our patients, have been documented (Table 1).^1^, ^2^, ^3^, ^4^, ^5^, ^6^, ^7^, ^8^, ^9^, ^10^, ^11^, ^12^, ^13^, ^14^, ^15^ The median age of the patients was 29 years (range, 1–73 years). Among the cases, 17 patients experienced gross hematuria, while three patients experienced thrombotic excretion and urinary retention. There has been no reported case of hematospermia. In several instances of post‐erectile hematuria, urethral hemangiomas were located in the posterior urethra. However, the hemangioma in our study was positioned between the verumontanum and external sphincter, and the patient exhibited symptoms of gross hematuria and urinary retention after ejaculation. Hemangiomas located in the anterior urethra sometimes appear as urethral bleeding, whereas lesions near the proximal urethra often lead to hematuria and urinary retention with blood clots.^13^ Larger lesions may cause urinary obstruction or protrude outward from the urethral opening.^13^ Cystourethroscopy emerges as a valuable tool for defining the location and extent of lesions, thereby simplifying pre‐surgery planning.^5^ Notably, Cattolica *et al*. have indicated that performing cystourethroscopy in a non‐erectile state may fail to detect urethral hemangioma lesions, which tend to enlarge and become more evident during the erectile state.^16^

**Table 1:** Clinical features of 23 patients with urethral hemangioma in males documented from 2000 to 2023

Case No	Age	Symptom	A relationship between symptom and male sexual activities	Size/location of hemangioma	Treatment	Published year	Authors (*et al*.)
1	Young	Hematuria	Unknown	—	Nd‐YAG laser	2000	Anurag K^1^
2	23	Painless hematuria	Unknown	10 × 10 mm/Bullbar urethra	Wide excision through a vertical penoscrotal	2001	Parshad S^2^
3	18	Hematuria	Unknown	22 × 9 mm/Bladder neck	Excision by cystostomy	2006	Mehmet D^3^
4	31	Hematuria, clot expulsion, urinary retention	Unknown	‐/Prostatic urethra	Transurethral removal with holmium laser	2008	Ponce J^4^
5	27	Urethral bleeding	Unknown	5 mm/5 cm far from the external urethral meatus	Simple transurethral excision	2009	Ioannis^5^
6	1	Hematuria	Not related	—	Transurethral resection	2011	Noviello C^6^
7	18	Urethral bleeding	Unknown	‐/6 cm far from the external urethral meatus	Surgical removing	2012	Maria A^7^
8	14	Urethral bleeding	Not related	5 mm/Pendular urethra	Transurethral ablation using Ho‐YAG laser	2013	Dig VS^8^
9	54	Hematuria	After ejaculation	‐/Prostatic urethra	Transurethral resection	2015	Han H^9^
10	39	Hematuria	After ejaculation	‐/Prostatic urethra	Transurethral resection	2015	Han H^9^
11	55	Hematuria	After ejaculation	‐/Prostatic urethra	Transurethral resection	2015	Han H^9^
12	44	Hematuria	After ejaculation	‐/Prostatic urethra	Transurethral coagulation	2015	Han H^9^
13	39	Hematuria	After ejaculation	‐/Prostatic urethra	Transurethral resection	2015	Han H^9^
14	73	Hematuria, urinary retention	After erection	‐/Prostatic urethra	Transurethral resection	2017	Hamada A^10^
15	41	Hematuria, painless urethral bleeding	Unknown	5 mm/Proximal penile urethra	Transurethral coagulation using monopolar electrocautery and Ho‐YAG laser	2017	Mohammad JS^11^
16	22	Hematuria	After erection	10 mm/Proximal penile urethra	Transurethral coagulation using Ho‐YAG laser	2017	Mohammad JS^11^
17	14	Painless urethral bleeding	Unknown	10 mm, 15 mm/Proximal penile urethra	Transurethral coagulation using Ho‐YAG laser	2017	Mohammad JS^11^
18	15	Hematuria	Unknown	‐/The membranous urethra	Injection Pingyangmycin	2019	Fang Y^12^
19	49	Painless hematuria, painless urethral bleeding	After erection	3 mm/2 cm away from the urethral meatus	Injection Pingyangmycin	2019	Fang Y^12^
20	18	Painless urethral bleeding	Unknown	‐/Anterior urethra distal to verumontanum	Cystoscopic fulguration using diathermy and injection Triamcinolone	2021	Afifa M^13^
21	6	Hematuria	Unknown	8 × 8 × 6 mm/prostatic urethra	Transurethral resection	2022	Alexandra MC^14^
22	64	Urethral bleeding	Unknown	7 mm/The navicular fossa	Thulium Fiber Laser for removing	2022	Genov P^15^
23	45	Painless hematuria, blood clots, urinary retention	After erection/ejection	6 × 6mm/prostatic urethra	Transurethral resection	2023	Our case

Saito *et al*. have proposed that conducting cystoscopy immediately after ejaculation with weakened perfusion enhances the likelihood of detecting hemangioma lesions.^17^ Our own experience aligns with their observations. We found that inducing an artificial erection and relaxing the external urethral sphincter muscle under general anesthesia effectively aids in identifying hemangiomas situated near the verumontanum during cystourethroscopy. Our observations during surgery have consistently shown clear visibility of hemangioma lesions (Fig. 1a,b). Furthermore, ultrasound of the penis and transrectal power Doppler sonography can often prove useful as they possess the ability to detect hypertrophic soft tissue and strong blood flow.^17^

Asymptomatic lesions typically do not require treatment, but a wide range of lesions often require incision, resection, and urethral reconstruction.^5^ Various treatment approaches have been suggested for urethral hemangiomas, including thermo‐ or laser coagulation for managing smaller lesions and abdominal surgeries for addressing larger lesions.^2^ However, these methods are not yet well established. In this study, we selected endoscopic resection using a high‐frequency electric knife because our clinic lacked the necessary laser equipment. To confirm the absence of bleeding and ensure complete removal of the hemangioma, we performed a digital rectal examination after resection, in alignment with findings from Hamada *et al*., who reported the detection of bleeding around the hemangioma via this method.^10^

Fang *et al*. reported that patients with urethral hemangioma in local hospital settings have received misdiagnoses such as seminal cystitis, urethritis, or prostatitis.^12^ Urologists should bear in mind that the most common symptoms associated with urethral hemangioma include intermittent hematuria and, frequently, indolence. Additionally, it is essential to recognize the potential for hematuria to develop after erection and ejaculation.

## Author contributions

Kimiyasu Ishikawa: Project administration; writing – original draft; writing – review and editing. Haruaki Sasaki: Data curation; funding acquisition; methodology; project administration; resources; validation. Hideaki Shimoyama: Investigation; validation. Motoki Yamagishi: Investigation; resources; validation. Hiroo Sugishita: Investigation. Yu Hashimoto: Investigation. Yuki Ichimura: Investigation. Ayana Doguchi: Investigation. Kazuhiko Oshinomi: Investigation; validation. Jun Morita: Investigation; validation.

## Conflict of interest

The authors declare no conflict of interest.

## Approval of the research protocol by an Institutional Reviewer Board

Not applicable.

## Informed consent

Written informed consent to participate in this study and for the publication of this report was obtained from the patient for ethics approval.

## Registry and the Registration No. of the study/trial

Not applicable.
